# Exposure of metal toxicity in Alzheimer’s disease: An extensive review

**DOI:** 10.3389/fphar.2022.903099

**Published:** 2022-08-29

**Authors:** Fahadul Islam, Sheikh Shohag, Shomaya Akhter, Md. Rezaul Islam, Sharifa Sultana, Saikat Mitra, Deepak Chandran, Mayeen Uddin Khandaker, Ghulam Md Ashraf, Abubakr M. Idris, Talha Bin Emran, Simona Cavalu

**Affiliations:** ^1^ Department of Pharmacy, Faculty of Allied Health Sciences, Daffodil International University, Dhaka, Bangladesh; ^2^ Department of Genetic Engineering and Biotechnology, Faculty of Earth and Ocean Science, Bangabandhu Sheikh Mujibur Rahman Maritime University, Dhaka, Bangladesh; ^3^ Department of Pharmacy, Faculty of Pharmacy, University of Dhaka, Dhaka, Bangladesh; ^4^ Department of Veterinary Sciences and Animal Husbandry, Amrita School of Agricultural Sciences, Amrita Vishwa Vidyapeetham University, Coimbatore, India; ^5^ Centre for Applied Physics and Radiation Technologies, School of Engineering and Technology, Sunway University, Subang Jaya, Malaysia; ^6^ Pre-Clinical Research Unit, King Fahd Medical Research Center, King Abdulaziz University, Jeddah, Saudi Arabia; ^7^ Department of Medical Laboratory Technology, Faculty of Applied Medical Sciences, King Abdulaziz University, Jeddah, Saudi Arabia; ^8^ Department of Chemistry, College of Science, King Khalid University, Abha, Saudi Arabia; ^9^ Research Center for Advanced Materials Science (RCAMS), King Khalid University, Abha, Saudi Arabia; ^10^ Department of Pharmacy, BGC Trust University Bangladesh, Chittagong, Bangladesh; ^11^ Faculty of Medicine and Pharmacy, University of Oradea, Oradea, Romania

**Keywords:** alzheimer’s disease, metal-induced toxicity, amyloid-beta, biometals, heavy metals, neurotoxicity

## Abstract

Metals serve important roles in the human body, including the maintenance of cell structure and the regulation of gene expression, the antioxidant response, and neurotransmission. High metal uptake in the nervous system is harmful because it can cause oxidative stress, disrupt mitochondrial function, and impair the activity of various enzymes. Metal accumulation can cause lifelong deterioration, including severe neurological problems. There is a strong association between accidental metal exposure and various neurodegenerative disorders, including Alzheimer’s disease (AD), the most common form of dementia that causes degeneration in the aged. Chronic exposure to various metals is a well-known environmental risk factor that has become more widespread due to the rapid pace at which human activities are releasing large amounts of metals into the environment. Consequently, humans are exposed to both biometals and heavy metals, affecting metal homeostasis at molecular and biological levels. This review highlights how these metals affect brain physiology and immunity and their roles in creating harmful proteins such as β-amyloid and tau in AD. In addition, we address findings that confirm the disruption of immune-related pathways as a significant toxicity mechanism through which metals may contribute to AD.

## GRAPHICAL ABSTRACT

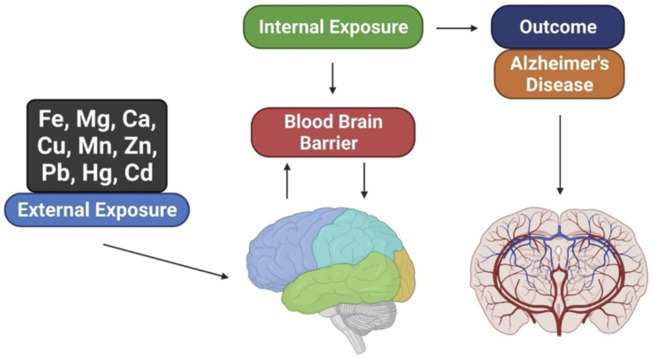

## Introduction

Alzheimer’s disease (AD) is a neurodegenerative disorder (NDD) that causes dementia in the elderly and has diverse implications (Islam et al., 2022b; 2022a). Neuropathological changes in the AD brain are linked to the aggregation of amyloid-β (Aβ) and the microtubule-associated tau protein in neurofibrillary tangles (NFTs), leading to cognitive impairment of neuronal connectivity and neuron loss (Kolarova et al., 2012). Aβ′s structure and harmful effects in causing oxidative stress (OS), autophagy, and neuroinflammation have been widely studied (Jomova et al., 2010; Uddin et al., 2020b). Several pharmacological candidates that remove or reduce Aβ production in AD treatment have been identified (Uddin et al., 2020a). In recent years, it has been determined that Aβ aggregation is not the initial event in AD pathogenesis but rather a later event (Kepp, 2017).

New research methodologies are required to develop successful AD treatments. According to various studies, homeostasis of key biometals such as calcium, magnesium, manganese, copper, zinc, and iron is disrupted in AD. Moreover, these metals play an important role in tau and Aβ metabolism and aggregation. It has been hypothesized that targeting metal interactions with Aβ may be effective in preventing AD (Li et al., 2017; Huat et al., 2019). The pathophysiological effects of metal imbalance in the brain have been established by several studies (Zhang Z. et al., 2016). Akhtar et al. found that intervention with chromium picolinate reduced streptozotocin-induced cognitive impairment (Akhtar et al., 2020). Furthermore, chromium picolinate treatment improved cognition and reduced oxidative damage, mitochondrial dysfunction and neuroinflammation, and increased insulin signaling, reversing AD pathophysiology. Nonetheless, some argue that impaired biometal activity is the cause of AD. The blood-brain barrier (BBB) makes treating brain disorders challenging. Because biometals cannot passively permeate the BBB, the metal imbalance in the AD brain cannot be attributed solely to decreased or increased exposure to metals but rather to a more complex initial intracellular ion distribution. Metal exporting, importing, and sequestering proteins maintain metal homeostasis in the brain (Harilal et al., 2020). Heavy metal accumulation in the human body harms various organs, particularly the brain. Several studies have focused on the neurological functions of cadmium, mercury, and lead in the brain (Kabir et al., 2021).

This review highlights the effects of biometals and heavy metals on the brain, including how they contribute to AD and immune system dysregulation. It also identifies treatment options for metal-induced neurotoxicity and important directions for future research.

## Pathogenic mechanisms of biometal–induced AD pathology

### Iron (Fe)

Iron (Fe) is an essential trace metal that causes oxidative damage and may contribute to NDD development. Several studies have shown a correlation between AD and oxidative damage (Levine, 1997). The putamen and globus pallidus have been found to contain higher iron levels in the brains of AD patients (Levine, 1997; Moon et al., 2016). However, serum iron levels in these AD patients were lower or unchanged compared to healthy individuals. The iron levels in the cerebrospinal fluid (CSF) were not affected by AD (Ficiarà et al., 2021). However, further research is required to confirm this observation due to the limited sample size of this CSF study.

Ferritin is an iron storage protein present at high levels in AD brain tissue (Quintana et al., 2006). Therefore, CSF ferritin may be a suitable measure of the amount of iron in the brain. Ferritin production (Thirupathi and Chang, 2019) and secretion (Zhang et al., 2006) by glial cells is dependent on cellular iron levels in cultured systems. CSF ferritin levels are thought to be representative of iron levels in the brain and can be useful in clinical settings. CSF ferritin levels are reduced in restless legs syndrome, a condition caused by low brain iron levels that are treated with iron supplements (Chawla et al., 2019). CSF ferritin levels were reportedly higher in AD patients (Kuiper et al., 1994). However, this observation was not confirmed in later studies with larger clinical cohorts (Paterson et al., 2014). Meta-analysis and cross-referenced statistical methods have been used to assess the iron content of twelve different regions of the brain. They found iron levels were higher in eight brain regions that were statistically linked to clinical AD diagnosis, and yellow blood iron levels and iron overload symptoms in the brain were present in AD patients whose iron homeostasis was unbalanced (Tao et al., 2014). However, a meta-regression analysis found that disparities in serum iron levels might result from differences in average participant age between trials (Wang et al., 2015).

**FIGURE 1:**
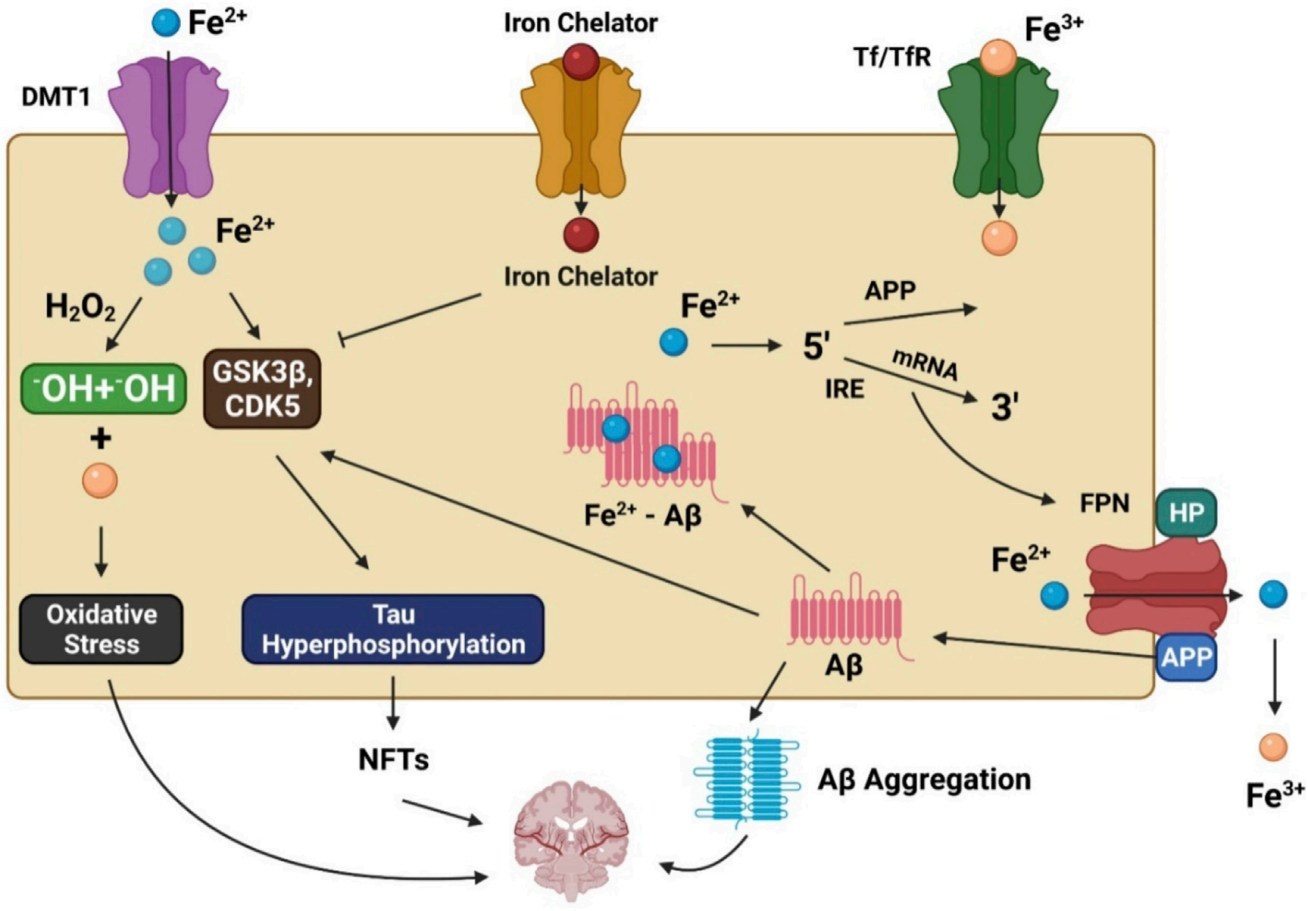
The involvement of iron in Alzheimer’s disease pathogenesis. DMT1 allows ferrous iron (Fe^2+^) to pass through the cell directly, while transferrin (Tf)-ferric iron (Fe^3+^) penetrates via endocytosis mediated by the transferrin receptor (TfR). Increased Fe^2+^ levels trigger the Fenton reaction, which produces the hydroxyl radical (•OH), resulting in oxidative stress and neurotoxicity. Moreover, Fe^2+^ can increase tau phosphorylation by activating glycogen synthase kinase 3β (GSK3β) and cyclin-dependent kinase 5 (CDK5), resulting in neurofibrillary tangle development (NFTs). GSK3 and CDK5 are inhibited by iron chelators, which diminish tau phosphorylation. Fe2+ interacts to the iron responsive element (IRE) in the 5′ UTR area of amyloid precursor protein (APP) mRNA in the biological environment, resulting in the stimulation of APP translation and the production of amyloid beta (Aβ).

Unfortunately, there is no well-supported explanation for these anomalies that lead to increased oxidative stress in AD patients because of their higher iron levels. For iron homeostasis to be maintained, there must be a dynamic interaction between iron efflux and influx in which many transporter proteins play a significant role. Iron accumulation in affected regions of the brain may be partially caused by dysfunction of the iron exporter ferroportin (FPN) and iron importers such as lactoferrin (Lf), melanotransferrin (MTf), divalent metal transporter 1 (DMT1), and transferrin (Tf) in AD patients (Figure 1). DMT1 and FPN are iron metabolism-related proteins involved in AD progression (Raha et al., 2014). *DMT1* is not expressed in oligodendrocytes or astrocytes. The Fe^2+^ influx process is associated with DMT1 (Song et al., 2007), which has two isoforms, DMT1+IRE and DMT1-IRE, that colocalize with Aβ in AD brain plaques. Additionally, levels of both isoforms were found to be increased in the hippocampus and frontal cortex of amyloid precursor protein (APP)/presenilin-1 (PSEN1) transgenic mice and associated with decreased *FPN* expression (Xian-Hui et al., 2015). The colocalization of FPN and hepcidin in astrocytes was associated with decreased *FPN* expression in the brains of AD patients. When hepcidin is repressed, the iron export process is inhibited, causing an iron buildup within cells (Raha et al., 2014). Studies have found that inhibition of APP-induced iron export reduces soluble tau levels leading to increased iron retention, which can be achieved using lithium (Lei et al., 2012, 2017) or an iron chelator (Lei et al., 2015). Moreover, sirtuin two regulates cellular iron homeostasis by deacetylating nuclear factor erythroid-derived 2-related factor 2, a transcription factor involved in the FPN synthesis regulation (Yang et al., 2017).

*DMT1* and *FPN* expression is reduced by chemicals present in Chinese herbs that may represent a novel approach for reducing iron overload-related impairment in AD patients (Xian-Hui et al., 2015). The Tf-transferrin receptor (TfR) complex facilitates iron uptake in BBB endothelial cells. Endocytosis of Tf-bound iron across the BBB can be facilitated by receptors, enabling iron transport. Significantly different CSF Tf levels were found in individuals carrying mutations than in relatives who did not have these mutations (Moos and Morgan, 2000; Ringman et al., 2012). Tf and Lf consist of two lobes, each with a Fe^3+^ binding site (Baker et al., 1994). *Lf* expression is elevated in macrophages and monocytes and fibrillar-type side population cells (SPs) in the cerebral cortex of AD patients. In addition, the aging process is mediated by SP creation. The endocytic process that eliminates Aβ is associated with the cell surface receptor lipoprotein receptor-related protein (LRP). Another beneficial function of Lf is its binding to LRP, dramatically improving the soluble amyloid removal instead of production (An et al., 2009; Wang et al., 2010). An Lf-based liposomal delivery method for neuron growth factors has been developed and used clinically, helping to prevent or reduce the spread of AD (Meng et al., 2015).

### Magnesium (Mg)

Magnesium helps keep intracellular calcium concentrations high under normal conditions by preventing calcium-induced excitatory responses (Levitsky and Takahashi, 2013). However, calcium and magnesium imbalances affect multiple processes that contribute to various health problems, including dementia (Volpe, 2013). Several studies have explored the effect of magnesium in AD pathogenesis. Hyperphosphorylated tau aggregation *in vitro* has been associated with magnesium deficiency (Yang and Ksiezak-Reding, 1999). Moreover, magnesium-l-threonate supplements reduce the enzyme β-secretase (BACE1), reducing levels of APP C-terminal fragments and free APP, reducing AD-associated cognitive impairment and synaptic loss (Li et al., 2014). Magnesium sulfate therapy reduces hyperphosphorylated tau levels by lowering glycogen synthase kinase 3 (GSK3) levels and blocking its phosphorylation, and enhancing phosphatidylinositol three kinase (PI3K) and protein kinase B (PKB) activity (Gomez-Ramos et al., 2006; Xu et al., 2014). Therefore, a neuroprotective magnesium effect may contribute to AD development.

Magnesium has been shown to alleviate chronic neuroinflammation by decreasing the calcium influx through N-methyl-d-aspartate receptors (NMDARs), a type of calcium-permeable cationic channel that contributes to the formation of long-term memories and learning activated by glutamate. Aggregation-induced NMDAR overactivation is observed in early-stage AD (Parameshwaran et al., 2008). There are two NMDAR subtypes identified in brain regions affected by AD with which magnesium interacts as an endogenous inhibitor (Kotermanski and Johnson, 2009).

Calcium influx into post-synaptic neurons was reduced by adding magnesium to block channels, reducing excitotoxic cell death in dementia. The activation of adenosine triphosphate (ATP)-gated P2X purinergic receptors (P2XRs) has also been associated with the onset of NDDs (Witting et al., 2004). Calcium enters and leaves cells through membrane pores formed by microglia using P2X7R oligomers (North, 2002). P2X7R and purinergic receptor-mediated neuroinflammation has been alleviated by magnesium in tissue culture, suggesting that increased magnesium levels may be an effective inhibitor of calcium entry via cell surface channels (Lee et al., 2011).

Transporters, exchangers, channels, and various buffering proteins maintain cellular calcium and magnesium levels. Magnesium transporter 1 (MagT1), cyclin M (CNNM) transporter, and transient receptor potential melastatin six and 7 (TRP-M6) enhance magnesium entry into cells. Magnesium release is through solute carrier family 41 member 1 (SLC41A1) and a sodium-independent magnesium exchanger (Romani, 2011; de Baaij et al., 2015). Several transporters, including calcium channels, are involved in maintaining intracellular calcium equilibrium. Calcium levels in the brain are increased by NMDAR, voltage-gated calcium channels, and store-operated channels. Buffer proteins such as calbindin can store calcium in the endoplasmic reticulum (ER), while the calcium-ATPase pump and sodium-calcium exchanger promote cellular calcium release. The AD brain’s ER-Ca dynamics are significantly affected by the activation of two types of calcium receptors and plasma membrane calcium-permeable channels (Tu et al., 2006; Cheung et al., 2008). However, the role of magnesium transporters in AD pathogenesis remains unknown. It was found that the physiological function of transient receptor potential cation channel subfamily M member 7 (TRPM7) is coordinated by presenilins, leading to familial AD (Oh et al., 2012). TRPM2 was removed from APP/PS1 mouse models to reduce ER stress and age-dependent memory impairments. In addition, *in vitro* studies showed that TRPM2 knockdown prevented an increase in the magnitude of the whole-cell current induced by Aβ, highlighting the importance of TRPM2 in the neuronal toxicity caused by Aβ. TRPM2 alterations have also been connected to calcium imbalance, despite their role in controlling magnesium associated with AD (Ostapchenko et al., 2015).

### Calcium (Ca)

Calcium has been identified as a widespread second messenger and regulator of cell functions (Komuro and Kumada, 2005). An *in vitro* study found it to contribute to the aggregation of hyperphosphorylated tau (Yang and Ksiezak-Reding, 1999). Calcium ion concentrations in the nervous system are tightly regulated by several mechanisms, including calcium channels, pumps, and binding proteins, and other metal ions such as magnesium. Magnesium has been found to be a calcium antagonist (Levitsky and Takahashi, 2013). There are a number of processes altered by calcium disturbances, including in NDDs such as AD (Volpe, 2013). Calcium-mediated neuroinflammation associated with NMDAR stimulation is reduced by magnesium through this pathway, preventing the long-term activation of the NMDAR-induced calcium influx. Because of their role in synaptic activities such as memory and learning, NMDARs are crucial calcium-permeable cationic channels. Overactivation of NMDARs by Aβ aggregation can occur in the early stages of AD (Parameshwaran et al., 2008). Continuous calcium influx can increase intracellular calcium concentrations, activating various enzymatic activities resulting in neuronal death, protein degradation, and oxidative stress (Mota et al., 2014). Several calcium transporters maintain intracellular calcium equilibrium. NMDARs and voltage-gated calcium channels are some of the receptors responsible for elevated calcium concentrations in the body. Calcium-ATPase pump and sodium-calcium transporter drive calcium release from cells, while buffer proteins such as calbindin store it in the ER. ER-Ca dynamics in the AD brain are significantly influenced by mutated presenilins, which activate two types of calcium receptors and plasma membrane calcium-permeable channels (Tu et al., 2006; Cheung et al., 2008). It has also been shown that Aβ oligomers can either activate calcium-permeable channels or bind to NMDARs, facilitating calcium influx into cells (Diaz et al., 2009; Arbel-Ornath et al., 2017).

### Aluminum (Al)

Aluminum is the third most abundant element in the earth’s crust and the most abundant metal. It is used in many applications, including food preservation, cans, cookware, automobiles, and vaccine adjuvants (Shaw and Tomljenovic, 2013). In mammals, specific functions of aluminum are obscure because of its toxicity to living organisms due to its strong reactivity with carbon and oxygen. The kidney quickly eliminates aluminum from food and environmental sources in humans. However, aluminum salts in vaccine adjuvants are biologically active and accumulate in the nervous system. Aluminum has been associated with AD and other NDDs (Aoun Sebaiti et al., 2018). It was found to accumulate with Aβ peptide in the brains of individuals with dialysis-associated encephalopathy (Ogunlade et al., 2022). Surprisingly, their symptoms disappeared soon after its removal from the dialysis solution (Exley and Mold, 2019). A recent meta-analysis found that chronic aluminum exposure increased the incidence of AD by almost 70% (Wang et al., 2016). Furthermore, an association between numbers of AD patients and their exposure to aluminum-adjuvanted vaccines was identified (Shaw and Tomljenovic, 2013), with increased levels of aluminum found in their hair, blood, and urine (Dórea, 2020). Aluminum hydroxide injections cause long-term memory loss, anxiety, and neurodegeneration in the spinal cord and motor cortex in mice (Shaw and Tomljenovic, 2013). Oxidative stress and mitochondrial dysfunction may also be responsible for neurological damage. However, several studies do not account for confounding factors such as genetic backgrounds that may predispose an individual to aluminum-induced neurological dysfunction. Aluminum-induced neurotoxicity is likely due to a combination of genetic and environmental factors (Wong-Guerra et al., 2021).

## Pathogenic mechanisms of heavy metal-induced AD pathology

### Copper (Cu)

The neurological system is very sensitive to heavy metals. Copper is an important transition metal involved in numerous biological processes, including energy metabolism and antioxidant defense. In addition, copper is involved in protecting against free radicals, cell respiration, and neurotransmitter synthesis (Zatta and Frank, 2007; Scheiber and Dringen, 2013). Copper deficiency may affect the production and maintenance of myelin causing neuronal degeneration (Table 1). In NDDs such as AD, copper levels are altered. However, the role of copper in AD remains enigmatic. Copper levels in senile plaques are abnormally high (Lovell et al., 1998). A deficiency in total copper in AD brain tissue has been observed in various studies (Deibel et al., 1996) and a recent meta-analysis that also found elevated plasma and serum copper levels in AD patients (Klevay, 2008). However, no significant difference in copper levels was observed in the CSF of healthy individuals and AD patients (Ventriglia et al., 2012; Vaz et al., 2018).

**TABLE 1:** Some heavy metal-induced Alzheimer’s disease-associated molecular objects.

Physical/chemical/clinical properties	Arsenic (As)	Lead (Pb)	Cadmium (Cd)	Mercury (Hg)
Absorption	Organic: also binds as trivalent and pentavalent>90%; inhalation: absorption is dependent on particle size; GI inorganic: trivalent and pentavalent salts >90%	Skin: alkyl lead compounds (methyl and tetraethyl lead) because of their lipid solubility; inhalation: up to 90% depending on particle size; GI: Adults have a GI of 5–10%, whereas children have a GI of 40%	Inhalation 10–40%; GI 1.5–5%	GI: inorganic salts can be absorbed and transformed to organic mercury by bacteria in the stomach; inhalation: elemental mercury is entirely absorbed
Distribution	Concentrates in the skin, nails, and hair; accumulates in the lungs, heart, kidney, liver, muscle, and brain tissue	Initially carried in red blood cells and dispersed to soft tissues (kidney and liver); primarily as a phosphate salt in bone, teeth, and hair	Binds to albumin and blood cells at first, then to metallothionene in the liver and kidney	Hg (vapor) penetrates membranes easily and quickly from the lungs to the CNS. Organic salts (lipid soluble) are equally distributed and eliminated by intestinal (intracellular) feces. Salts that are inorganic concentrate in the blood, plasma, and kidneys (renal elimination)
Half-life	7–10 h	Blood: 30–60 days; bone: 20–30 years	10–20 years	60–70 days
Sources of exposure	GI: food and well water Environmental: smelting ore waste, such as Ga in semiconductors, herbicides, and insecticides; inhalation: smelting fumes and dust	GI: paint, pottery, moonshine; inhalation: metal fumes skin: tetraethyl lead in gasoline	Inhalation: industrial, metal fumes, tobacco; environmental: electroplating, galvanization, plastics, batteries; GI: pigments, polishes, antique toys	Environmental: electronics and plastic industry; seed fungicide treatment; dentistry
Mechanism of toxicity	Membranes: Capillary endothelium protein damage increased vascular permeability, resulting in vasodilation and vascular collapse; inhibition of sulfhydryl group containing enzymes; suppression of anaerobic and oxidative phosphorylation (substitutes for inorganic phosphate in synthesis of high-energy phosphates)	Heme production is inhibited; heme is a key structural component of hemoglobin, myoglobin, and cytochromes		
Binds to proteins’ sulfhydryl groups (-SH groups)	Inhalation: emphysema, local irritation, and suppression of alpha1-antitrypsin; oral: kidney: proximal tubular damage (proteinuria) linked to beta2-acroglobulin	Protein precipitation and destruction of mucosal membranes due to salt dissociation; necrosis of the proximal tubular epithelium; inhibition of sulfhydryl (-SH) group containing enzymes		
Treatments	Exclusion from exposureAcute: supportive therapy: fluid, electrolyte replacement, blood pressure support (dopamine); chronic: penicillamine w/o dialysisrsine gas (AsH_3_) acts as a hemolytic agent with secondary to renal failure. Supportive therapy: transfusion; chelators have not been proved to be effective	Treatment with chelators such as CaNa2EDTA, BAL, dimercaprol, and d-penicillamine after removal from exposure	Removal from exposure, chelation therapy using CaNa2EDTA, and BAL, although the BAL-Cd combination is exceedingly toxic and is not utilized	Removal from exposure; Hg and Hg salts >4 *μ*g/dl: 2,3-dimercaptopropanol (BAL), *β*, *β*-dimethyl cysteine (penicillamine), most effective is N-acetyl-*β*, *β*-dimethyl cysteine (N-acetyl-penicillamine); methyl Hg-supportive treatment (nonabsorbablethiol resins can be given orally to reduce methyl Hg level in gut)
Humans’ maximum allowable dosage	10–50 *μ* gkg ^−1^ (EPA References)	5 *μ* gkg ^−1^day ^−1^ (EPA References)	0.5–1 *μ* gkg ^−1^day ^−1^ (EPA References)	0.1–2 *μ* gkg ^−1^day ^−1^ (EPA References)
References	(Yadav et al., 2010; Sharma et al., 2014)	(Sharma et al., 2014; Kumar Singh et al., 2018)	(Sharma et al., 2014; Batool et al., 2019)	(Sharma et al., 2014; Patel and Rao, 2015)

Copper precipitates in large amounts in senile plaques, causing copper insufficiency elsewhere in the body. Copper has been shown to negatively influence the pathological mechanisms of Aβ and tau (Sparks and Schreurs, 2003; Kitazawa et al., 2009), potentially explaining this heterogeneity. Copper has a strong affinity for Aβ and stimulates the production of its oligomers (Tõugu et al., 2008; Jin et al., 2011). Because copper and Aβ can catalytically produce hydrogen peroxide *in vitro*, oxidative stress may be a factor in copper-mediated Aβ oligomer cytotoxicity. Copper chelators such as clioquinol can counteract Cu-Aβ toxicity (Adlard et al., 2008; Matlack et al., 2014). Copper is also present in A precursor-like protein 2 (APLP2) and APP (Barnham et al., 2003). Indeed, higher copper levels in the cerebral cortex of mice missing APP or APLP2 indicate that the copper transporter APP may also function as a chelator (White et al., 1999b). However, another study by the same authors found that APP deletion in cortical neurons had no impact on copper uptake (White et al., 1999a), suggesting that they may not be a copper carrier but instead reflect improper copper interactions.

Copper increases exocytosis and decreases endocytosis, facilitating the redistribution of APP to the cell membrane (Acevedo et al., 2011). Copper has also been shown to enhance the GSK3-mediated dephosphorylation of endogenous APP and facilitate its proteolytic degradation into Aβ (Acevedo et al., 2014). In addition, the binding of copper to the microtubule-binding domain of tau causes its aggregation *in vitro* (Su et al., 2007). Hydrogen peroxide production was induced by copper exposure in a mouse AD model (Kitazawa et al., 2009). The activation of cyclin-dependent kinase 5 (CDK5) and GSK3 pathways is believed to be how copper mediates tau phosphorylation (Crouch et al., 2009).

Copper trafficking mechanisms in the AD brain are well understood. P-type ATPases, particularly ATP7A and ATP7B, play a crucial role in controlling monovalent copper in cells together with the transporters, high-affinity copper uptake proteins 1 (CTR1) and 2 (CTR2) (Kuo et al., 2006; Yu et al., 2017). Copper-containing enzymes are synthesized by DMT1 in cells that receive divalent copper from DMT1 (Zheng and Monnot, 2012). However, ATP hydrolysis can reduce copper overload in cells, and this process is used by both ATP7A and ATP7B to export copper from cells.

**FIGURE 2:**
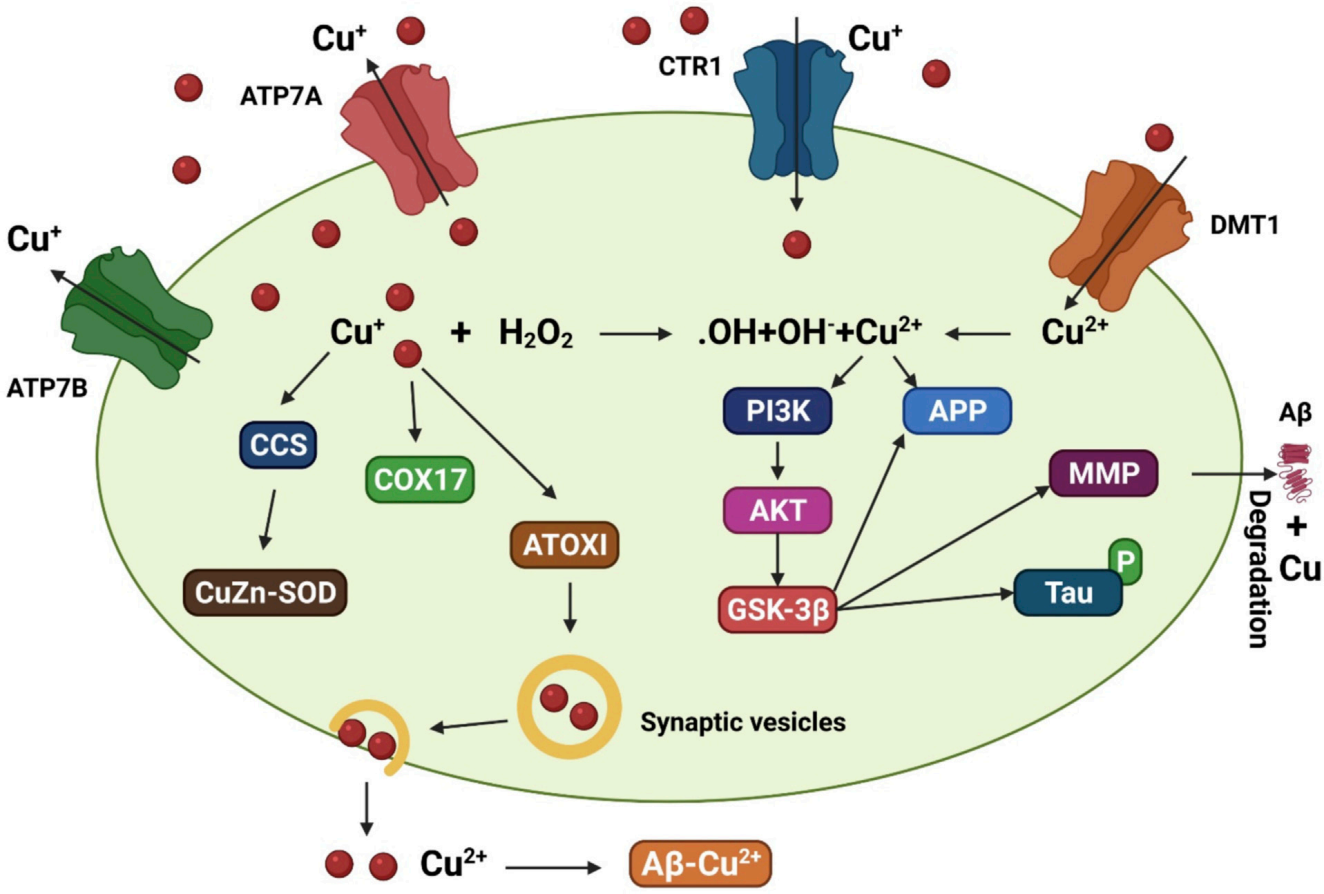
A model that depicts the copper transit system and its link to AD. Copper transporter 1 (CTR1) transports Cu^+^ into brain cells. Cu^2+^ uptake is aided by DMT1. Various Cu chaperones, including such copper chaperone for superoxide dismutase (CCS), cytochrome oxidase enzyme complex (COX17), and antioxidant protein (ATOX1), sequester accumulated Cu into particular cellular sites. ATOX1 is thought to transfer Cu^+^ to ATP7A (copper-transporting P-type ATPase) and ATP7B, which aid in the import of Cu^+^ into synaptic vesicles for release and/or facilitate Cu export straightforwardly. Increased oxidative stress may be caused by excessive intracellular Cu^+^ activating the Fenton reaction. Cu^2+^ also leads to tau hyperphosphorylation by stimulating the glycogen synthase kinase 3β (GSK3β) pathway, which is implicated in the production of the matrix metalloproteinases (MMP) important for Aβ breakdown. Copper interacts to Aβ in the synaptic cleft, facilitating the production of senile plaques.

In addition to transporters, many molecular chaperones such as antioxidant protein 1, the enzyme complex cytochrome oxidase, and copper chaperone for superoxide dismutase (SOD) contribute to copper delivery (Harris, 2001). Studies found that genetically deleting the copper transporter 1C (CTR1C) gene, a member of the CTR1 family with high homology to its human ortholog, in a *Drosophila* AD model drastically decreased levels of copper accumulation in the brain (Lang et al., 2013). In addition, when another copper importer, copper transporter 1B (CTR1B), was suppressed or when the copper exporter ATPase copper transporting 7 (ATP7) gene was overexpressed in this AD model, the same outcome was observed (Figure 2). Flies with CTR1 knocked down had higher levels of Aβ production but lower levels of oxidative stress, suggesting that increased Aβ oligomers or Aβ aggregates are less harmful with reduced copper influx (Lang et al., 2013). When amyloid plaques are present, ATP7-alpha (ATP7A) levels are increased in nearby activated microglial cells, leading to a dramatic shift in copper trafficking. AD can be associated with inflammation-induced copper dyshomeostasis in microglia based on this neuromechanism (Zheng et al., 2010). An accumulation of single nucleotide polymorphisms in the ATP7-beta (*ATP7B*) gene is associated with an increased risk of AD development, indicating that changes in copper homeostasis may accelerate AD-associated neurodegeneration (Bucossi et al., 2011; Squitti et al., 2013).

### Manganese (Mn)

Manganese is an essential trace element contributing to the growth of human tissues and the regulation of intracellular homeostasis (Prakash et al., 2017). SOD and glutamine synthetase are two important manganese-dependent enzyme cofactors. There is increasing evidence that manganese overload is associated with NDDs and that even a slight manganese excess can cause symptoms that are comparable with manganese (Park et al., 2014). This cell toxicity is caused by various mechanisms, including oxidative stress and mitochondrial dysfunction, abnormal energy metabolism, toxic chemical accumulation, cellular depletion of antioxidant defenses, and autophagy (Guilarte, 2013; Martinez-Finley et al., 2013). Manganese levels in the brains of AD patients with dementia were found to be significantly higher than in healthy individuals, with the highest concentrations in the parietal cortex (Srivastava and Jain, 2002; Tong et al., 2014). Plaques were disseminated in monkeys exposed to chronic manganese levels. The p53 pathway targets the most affected gene in the frontal cortex, amyloid-beta precursor-like protein 1 (APLP1) (Guilarte, 2010). The frontal cortex appears to be a primary target of manganese exposure, contributing to early dementia (Schneider et al., 2013). The mechanism by which manganese treatment elevates Aβ peptide levels is likely related to the disruption of Aβ degradation (Tong et al., 2014). A recent study found, similar to other biometals, manganese can weakly bind to a specific region of Aβ (Wallin et al., 2016). Additional research is required to fully understand these initial findings and determine how manganese binding to Aβ affects Aβ aggregation.

The antioxidant enzyme Mn-SOD contains manganese and is crucial for maintaining mitochondrial health. Oxidative respiration is inhibited by increased manganese, increasing reactive oxygen species (ROS) generation and mitochondrial dysfunction (Gunter et al., 2006). Aβ plaque deposition and tau phosphorylation in a transgenic AD mouse model were elevated when Mn-SOD was partially inhibited (Li et al., 2004; Melov et al., 2007). However, the overexpression of Mn-SOD reduced the load of cortical plaques associated with AD pathology (Dumont et al., 2009), associating AD pathogenesis with mitochondrial oxidative stress. Because manganese and iron compete to some extent for binding sites and transport channels in the Golgi apparatus, it has been hypothesized that excessive manganese absorption causes Golgi iron deficiency (Carmona et al., 2010).

**FIGURE 3:**
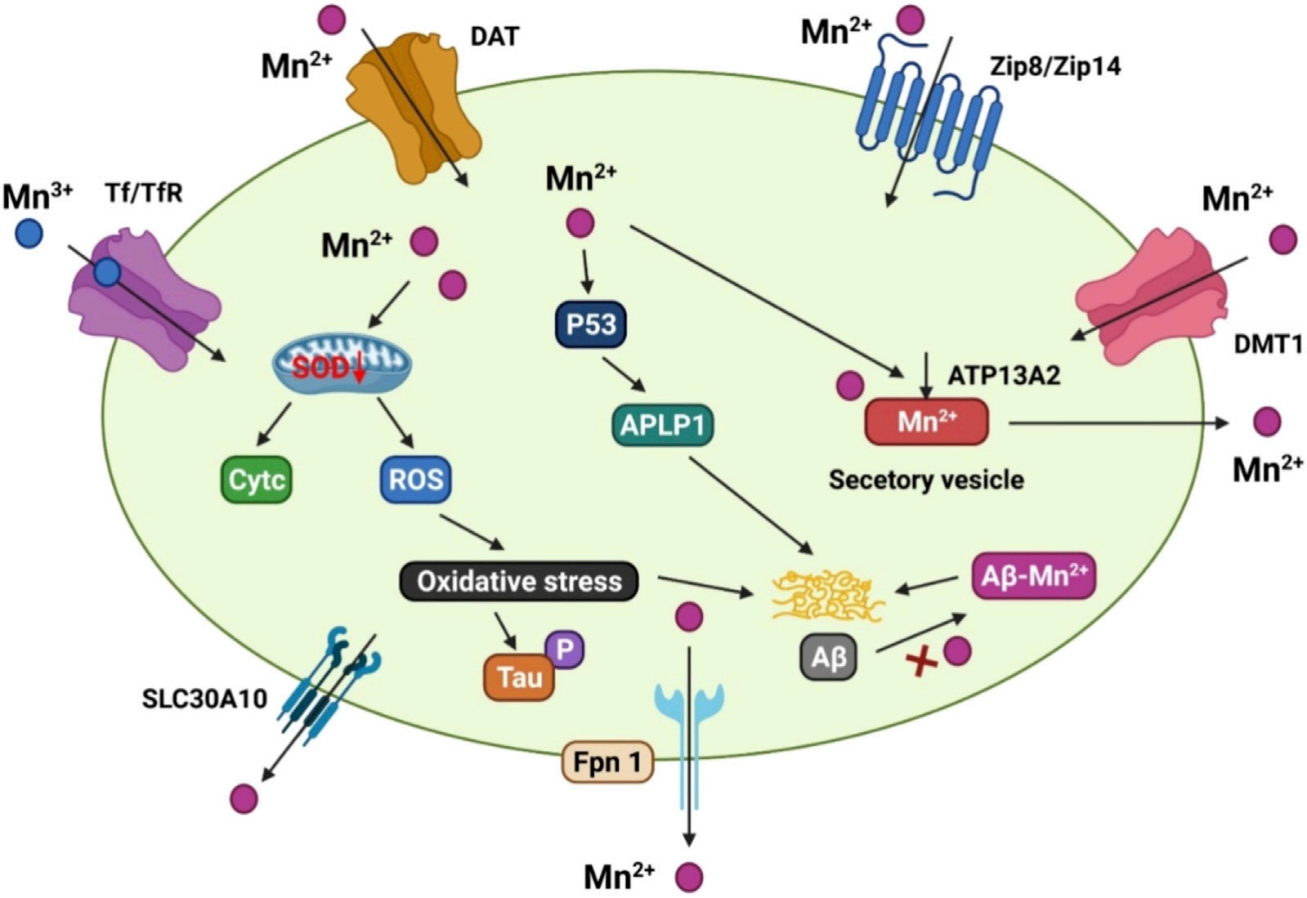
The manganese transport mechanism, and its association with Alzheimer’s disease. DMT1, ZIP8/ZIP14, and dopamine transporter (DAT) are involved for Mn^2+^ inflow on the cell membrane, whereas Tf/TfR mediates Mn^3+^ entrance into the endosome via endocytosis and is then released into the cytoplasm by DMT1. SLC30A10 and Fpn, on the other hand, transport Mn^2+^ out of cells. ATP13A2 and SPCA1 also transport Mn^2+^ into the lysosomes and Golgi for bioavailability, or produce secretory vesicles that aid Mn^2+^ efflux. Mn^2+^ conditions can cause mitochondrial oxidative stress in the AD brain, which accelerates tau phosphorylation. In addition, elevated Mn^2+^ levels boost the production of p53 and its transcriptional target gene, amyloid-b precursor-like protein 1 (APLP1), which encodes amyloid precursor protein (APP). The production of Aβ peptides is aided by enhanced APLP1 expression. Mn^2+^ could potentially attach to Aβ and help its aggregation.

Manganese transport is mediated by numerous importers, such as the dopamine transporter (DAT), DMT1, Tf/TfR, zinc transporters 4 (ZIP4) and 8 (ZIP8), secretory route Ca^2+^-ATPase 1 (SPCA1), ATPase cation transporting 13A2 (ATP13A2/PARK9), solute carrier family 30 member 10 (SLC30A10), and FPN (Figure 3) DMT1 was the first mammalian transporter for cellular manganese absorption and an iron influx transporter. When iron is scarce, DMT1 facilitates the efficient transfer of manganese across the BBB (Garrick et al., 2006). Trivalent manganese enters cells by ligand-receptor endocytosis, while divalent manganese enters cells via DMT1 (Subramaniam et al., 2002). Zinc-binding ZIP8 and zinc transporters 14 (ZIP14) have been found by multiple studies to contribute to manganese absorption in the liver and lungs (Aydemir et al., 2017; Lin et al., 2017).

Recent studies have focused on the role of exporter proteins in maintaining manganese levels. The cell surface-located efflux exporter SLC30A10 was identified in genome research to be a potential zinc and manganese transporter. There is an increased accumulation of manganese in the brain of Parkinson’s disease patients who carry SLC30A10 mutations (Tuschl et al., 2016). In the frontal cortex of AD patients and APP/PS1 transgenic mice, SLC30A10 levels are consistently lower, indicating that its dysregulation may be a contributing factor to the AD onset and progression (Bosomworth et al., 2013).

Several studies have shown that the iron exporter FPN operates as a cellular manganese exporter in a pH-dependent manner to reduce manganese buildup and cytotoxicity in the body (Madejczyk and Ballatori, 2012). The ATP13A2 cation transporter transports manganese and zinc, among others. Overexpression of ATP13A2 has been shown to lower intracellular manganese concentrations, lowering manganese-induced mortality. ATP13A2 loss-of-function mutations are associated with increased α-synuclein and Aβ plaques in Lewy body dementia (Murphy et al., 2014).

SPCA1 homolog calcium-transporting ATPase 1 (PMR1) has been shown to mediate calcium and manganese transport, and the ectopic expression of SPCA1 in yeast increases their susceptibility to manganese poisoning (Ton et al., 2002). While SPCA1 has been proposed as a secondary regulator of cellular manganese homeostasis, the affinity between SPCA1 and manganese and the roles of SPCA1 in AD pathogenesis requires further research.

### Zinc (Zn)

The brain is the organ with the highest concentration of zinc in the body. Zinc-dependent transcription factors and enzymes represent more than 2,000 proteins in the brain, and zinc is found in 70% of all brain proteins studied so far (Takeda, 2000). Zinc is transported into the brain parenchyma across the BBB and CSF barriers. For example, zinc can be transported across the BBB through its interaction with l-histidine in both plasma and CSF (Takeda, 2001). In the CSF and extracellular fluid compartments, zinc can be readily exchanged following its uptake by the body (Takeda, 2001). One of the three families of proteins that regulates zinc homeostasis in the brain is the zinc-binding proteins (ZBPs), which are primarily responsible for regulating intracellular zinc levels (Mocchegiani et al., 2001; Vanea et al., 2014; Islam et al., 2022c, 2022c, 2022c). Zinc uptake from extracellular fluids into neurons and glia is regulated by zinc and iron-like regulatory proteins, while zinc outflow from cells is regulated by zinc transporters (Liuzzi and Cousins, 2004; Kambe et al., 2015). Interestingly, many zinc regulatory proteins also regulate other metal anions.

Glutamatergic nerve terminals in the brain are particularly rich in zinc ions, which can be released into the environment during neuronal activity (Paoletti et al., 2009; Sensi et al., 2011). Synaptic zinc release influences the development and function of glutamatergic receptors such as NMDA and receptors for glycine ionotropic and gamma-aminobutyric acid (GABA) and other neurotransmitters (Smart et al., 2004). Therefore, zinc is crucial for memory and behavior since it is associated with the balance between excitation and inhibition in the brain (Frederickson and Danscher, 1990).

There is a broad spectrum of neurological disorders caused by the disruption of zinc homeostasis (Doraiswamy and Finefrock, 2004; Mocchegiani et al., 2005). While zinc lacks redox activity, high zinc levels in the extracellular fluid have been shown to cause neurotoxicity and alter protein aggregation (Cuajungco and Lees, 1997; Frederickson et al., 2005). The discovery that zinc can precipitate Aβ into plaques at concentrations >300 nM generated interest in its function in AD (Bush et al., 1994). Synaptic transmission boosts the concentration of zine in the extracellular fluid, potentially explaining why Aβ deposition occurs in the brains of AD patients (Deshpande et al., 2009). Plaques and the cerebral amyloid angiopathy around affected blood vessels contain zinc-rich metalloprotein Aβ, itself a metalloprotein containing zinc binding sites (Bush et al., 1993; Suh et al., 2000; Maynard et al., 2005). The increase of Zn^2+^ in presynaptic vesicles in Tg2576 transgenic mice crossed with zinc transporter three knockout mice have been shown to reduce plaque load, demonstrating that synaptic zinc contributes to Aβ deposition (Lee et al., 2002). When zinc is sequestered by Aβ, it inhibits APP ferroxidase function, resulting in increased iron and ROS levels (Mucke et al., 2000; Duce et al., 2010).

Extrinsic zinc-chelator CEDAA combined with cadmium can reverse the attenuation of long-term potentiation (LTP) in dentate granule cells produced by Aβ and zinc treatment in dentate granule cells *in vivo* (Takeda et al., 2017). Consequently, zinc chelators or ionophores can restore the physiological metal ions sequestered inside extracellular Aβ aggregates, resulting in biochemical and anatomical alterations that contribute to improved cognition (Adlard et al., 2008, 2011, 2015). Despite the overwhelming evidence that zinc and copper chelators minimize Aβ accumulation, it has recently been shown that this may have unintended negative consequences for brains in good health (Adlard et al., 2008). The zinc/copper chelator clioquinol decreased memory function in young mice (2.5 months old) by depleting zinc levels in their brains. The brain-derived neurotrophic factor (BDNF) associated with synaptic plasticity and dendritic spine density has been shown to increase *in vivo* (Frazzini et al., 2018). The hippocampus, cortex, and striatum were all affected, but the cerebellum, which is devoid of zinc reservoirs, was unaffected (Wall, 2005; Frazzini et al., 2018). The role of zinc in promoting its effects, particularly at the cellular and molecular levels of the brain, remains to be studied in greater detail.

### Lead (Pb)

One of the most common names for the heavy metal plumbum (Pb) is lead. While the dangers of lead poisoning had been understood for many years, the association between white lead paint on porches and railings in Brisbane, Australia, and severe neurological abnormalities in children was not recognized until 1892 (Needleman, 2009). The half-life of environmental lead in the bloodstream is 30 days. Lead attaches to blood cells and travels throughout the body, eventually accumulating in the bones. Bone-deposited lead has a half-life of 20–30 years. Bone demineralization during pregnancy, menopause, lactation, and aging induces the release of accumulated lead into the bloodstream (Nash et al., 2004; Rastogi et al., 2007).

Many systems in the body are affected by lead in the blood, but the central nervous system is by far the most vulnerable. The effects of lead on the brain can be divided into two categories: morphological and pharmacological. Neuronal differentiation, myelination, and synaptogenesis are examples of morphological effects (Brubaker et al., 2009; Hu et al., 2014; Senut et al., 2014). Biometal-dependent systems can be disrupted due to binding site competition between lead and other biometals, particularly calcium and zinc. Lead rapidly crosses the BBB and severely damages the brain due to its capacity to substitute for calcium ions (Sanders et al., 2009). The GABAergic, dopaminergic, and cholinergic systems and NMDA receptors are inhibited by lead, interfering with neurotransmitter release. Additionally, lead binds to sulfhydryl groups in glutathione, a crucial antioxidant present in cells, removing its antioxidant properties (Bijoor et al., 2012; Flora et al., 2012).

Lead exposure during childhood has been shown to cause cognitive and behavioral problems (Mazumdar et al., 2011; Reuben et al., 2017). Neuronal alterations in hippocampal CA1 pyramidal neurons associated with memory loss and learning impairments are associated with juvenile lead exposure. Animals exposed to lead while pregnant or after birth suffer from memory loss and cognitive decline in old age (Hu et al., 2008; Bihaqi et al., 2014). Low-level gestational lead exposure has been shown to change the hippocampus dendritic spines by decreasing neuroligin-1 protein levels, resulting in memory and learning problems (Zhao et al., 2018). A probable link between AD development and the long-term effects of lead exposure in childhood has been suggested. According to a long-term study of former organolead production workers, lead exposure causes cognitive impairment over time and leaves permanent brain damage (Schwartz et al., 2000; Stewart et al., 2006).

Lead has been associated with a number of AD hallmarks, including Aβ buildup, tau pathology, and inflammation. Moreover, early lead exposure caused an addiction-like disease in young rats, resulting in increased *APP* and *BACE1* expression, and inducing Aβ buildup and plaque development in the hippocampus and cortex, respectively (Zhou et al., 2018). *APP* and *BACE1* expression were found to be upregulated in aged rat brains following prenatal exposure to lead (Basha et al., 2005). Lead exposure in childhood boosted the expression of *APP*, *BACE1*, and transcription factor-specific protein 1 (*Sp1*) to a similar extent and facilitated Aβ deposition in elderly monkeys (Wu et al., 2008). Lead, cadmium, and arsenic exposure synergistically increased *APP* and *BACE1* expression, strongly inducing Aβ production (Ashok et al., 2015). Exposure to lead during development activates the sterol regulatory element-binding protein 2 (SREBP2)-BACE1 pathway, affecting normal cholesterin metabolism in the early brain (Wu et al., 2008).

It has been well established that dysregulation of cholesterol homeostasis in the brain plays a significant role in the genesis of AD and Aβ production (Maria Giudetti et al., 2015). Acute lead exposure has been shown to enhance Aβ accumulation in the brain tissue and CSF by disrupting low-density lipoprotein receptor-related protein 1 (LRP-1)-mediated clearance of the peptide (Gu et al., 2011). Notably, lead exposure also increases levels of total and hyperphosphorylated tau. It has been shown that lead exposure increases the protein levels of tau and phosphorylated tau in SH-SY5Y neuroblastoma cells (Bihaqi et al., 2017). Tau expression and serine/threonine phosphatase and CDK5 activities in the brain are all up-regulated following lead exposure early in life (Bihaqi and Zawia, 2013). GSK3 and caspase-3-mediated tauopathy have recently been associated with lead exposure (Bihaqi et al., 2018).

Neuronal death occurs as a result of an inflammatory response to lead exposure. Tumor necrosis factor-alpha (TNF-α) and granulocyte-colony stimulating factor levels are higher in individuals exposed to lead than in those who were not (Di Lorenzo et al., 2007). The persistent stimulation of glial cells in a rat model was accompanied by inflammation and neurodegeneration. Microglia are activated and pro-inflammatory proteins such as inducible nitric oxide synthase (iNOS), interleukin one β (IL-1β), and TNF-α. AD-related brain neurotoxicity is thought to be caused in part by the substances listed above. Lead exposure leads to increased microglial activation and poorer LTP (Liu et al., 2012). The activation of the transcription factor nuclear factor kappa B (NF-κB) and the overexpression of cyclooxygenase-2 is the cause of lead-induced activation of microglia. Other microglial pathways associated with lead exposure include extracellular signal-regulated kinase (ERK) and PKB activation (Kumawat et al., 2014). Additionally, lead exposure was found to stimulate TLR4-MyD88-NF-κB signaling, affecting hippocampal neurogenesis and plasticity (Liu et al., 2015). Lead activation results in increased synthesis of proinflammatory cytokines and generation of reactive nitrogen species (RNS) and ROS (Altmann et al., 1999). These findings conclusively show that long-term lead exposure raises AD risk. However, in the absence of medications to counter lead poisoning, exposure should be avoided.

### Mercury (Hg)

AD has been associated with mercury exposure. Numerous studies have found elevated mercury levels in the blood and brain tissue of AD patients (Ehmann et al., 1986; Thompson et al., 1988; Hock et al., 1998). Consequently, mercury levels have been found to be higher in hair samples. In a comparison of patients with and without degenerative brain diseases, mercury levels in patients with degenerative brain diseases were higher than in patients without (Mano et al., 1989). Indeed, mercury in the neurological system has been found to cause memory loss, attention problems, and even dementia, a common indicator of AD (Zahir et al., 2005; Mutter et al., 2010).

### Cadmium (Cd)

Cadmium is an abundant heavy metal in the environment and a naturally occurring carcinogen that causes cancer in humans. Unlike other heavy metals, cadmium is water-soluble, allowing it to be transmitted from the soil to plants and accumulate in the food chain (Qadir et al., 2014; Laslo et al., 2022, 2022). For example, tobacco can tolerate high levels of cadmium despite its potentially harmful effects on other plants (Huang and Goldsbrough, 1988; Zhang M. et al., 2016). Consequently, the general public’s risk for cadmium-related morbidities is increased by their use of tobacco products or inhalation of tobacco smoke (Richter et al., 2017).

Cadmium has a half-life of 20–40 years in the kidneys and liver after entering the body (Suwazono et al., 2009; Fransson et al., 2014). Cadmium has been shown to pass through the BBB and accumulate in the brain, causing neurotoxicity (Wang and Du, 2013). Older individuals with high blood cadmium levels were found more likely to die from AD-related causes (Wang and Du, 2013; Min and Min, 2016). A growing body of evidence suggests that cadmium may contribute to the aggregation of Aβ plaques in AD patients (Li et al., 2012). Cadmium administration to APP/PS1 mice caused an increase in plaque size and quantity *in vivo* (Li et al., 2012). Cadmium ions can promote plaque development through their interaction with Aβ (Notarachille et al., 2014). Cadmium therapy has also been proposed to suppress the expression of β-secretase and neutral endopeptidase, which are both important for decreasing Aβ levels in the brain (Li et al., 2012). The synergistic effect of cadmium, lead, and arsenic greatly improves amyloidogenesis by increasing *APP*, *BACE1*, and *PSEN1* expression, suggesting that cadmium interacts with other metals in AD (Ashok et al., 2015).

In addition to its effects on Aβ, cadmium has been implicated in tau conformation and self-aggregation in the AD brain (del Pino et al., 2016). The third repeat (R3) of the microtubule-binding domain of tau has been shown to bind to cadmium. Tau self-aggregation is facilitated by the loss of the random coil conformation and gain of a helix shape of the R3 domain. Cadmium therapy suppresses muscarinic M1 receptors, known to adversely regulate GSK3 and increase levels of total and phosphorylated tau (Medeiros et al., 2011; del Pino et al., 2016). These findings are consistent with the view that cadmium may play a role in AD development.

Nontoxic cadmium exposure has been shown to activate the MAPK and NF-κB signaling pathways, leading to neuroinflammation and neuronal death in humans (Phuagkhaopong et al., 2017). Increased expression of interleukins 6 (IL-6) and 8 (IL-8) has been associated with AD development. The MAPK and PI3K/AKT signaling pathways have also been found to contribute to cadmium cytotoxicity in astrocytes (Jiang et al., 2015). Therefore, central nervous system (CNS) illnesses such as AD and Parkinson’s disease may be prevented by controlling cadmium-induced Ca^2+^ homeostasis. However, adverse neuroinflammation has yet to be associated with cadmium exposure in animal AD models.

## Neurotoxicity induced by metal mixtures

Most studies of metal neurotoxicity have focused on one specific metal. However, the reality is that we live in a world where metals coexist, making research more complex. Since numerous metals such as iron, manganese, copper, cadmium, and zinc are transported or controlled by overlapping signaling pathways, fluctuations in the levels of one metal can significantly impact the homeostasis of another. The combined effects of exposure to multiple metals have been rarely studied. However, cadmium, mercury, and manganese have been studied in the context of lead neurotoxicity (Sanders et al., 2015).

Prenatal exposure to lead is more harmful to brain development than prenatal exposure to just one metal. Children exposed to high levels of cadmium during pregnancy may experience problems with their mental and motor development if their lead levels are also high (McDermott et al., 2011; Kim et al., 2013; Sanders et al., 2015). In addition, prenatal exposure to high levels of both lead and manganese led to a greater number of cognitive and language impairments in infants by the age of two compared to exposure to each alone (Lin et al., 2013). Furthermore, lead exposure was associated with poorer IQ scores in children with high blood manganese levels, but only if they also had high blood manganese levels. Rather than functioning cooperatively, lead and arsenic exposure appears to function noncooperatively since their combined effects on cognitive deficiency were not additive, contrary to the findings with lead and cadmium, manganese, or mercury exposure (Kim et al., 2009; Yorifuji et al., 2011).

The retention and redistribution of individual metals in rodents can be improved by the co-administration of two metals (Kalia et al., 1984). Chandra et al. gave rats lead intraperitoneally along with manganese orally and found they experienced more severe changes in motor activity, learning ability, biogenic amine, and brain lead levels than rats given manganese or lead alone (Chandra et al., 1983). The central monoaminergic systems of mice have been found to be affected by an interaction of arsenic/lead. While single metal treatments lowered norepinephrine in the hippocampus, combined treatments of arsenic and lead increased serotonin levels in the midbrain and frontal cortex and lead accumulation in the brain (Mejía et al., 1997). When rats were fed mining waste containing arsenic, cadmium, manganese, and lead orally, arsenic and manganese were shown to accumulate in the brain, and dopamine release was reduced over time with LTP.

Manganese has been shown to exacerbate the well-documented neurotoxic effects of lead in children at younger ages (Rodríguez et al., 1998; Sanders et al., 2015). Rat motor parameters were significantly reduced after exposure to a mixture of arsenic, manganese, and lead rather than a single metal alone. Because humans are frequently simultaneously exposed to multiple metals in the real world and metal overexposure is frequently associated with neurotoxicity, additional research into the health effects of mixed metal exposure is urgently needed in the near future.

## Treatment for metal-induced neurotoxicity

The initial steps in treating individuals poisoned by metals are to remove them from the hazardous area and then perform gastrointestinal decontamination. For example, a fast fall in blood plasma manganese levels was observed after manganese supplementation was discontinued in children with cholestasis receiving total parenteral nourishment (Hambidge et al., 1989). Plasma-based therapy may be ineffective in individuals with chronic metal exposure because the metals will already have accumulated in the bones, brain, and other tissues. Chelation therapy, in which multiple metals are removed from the body, is a common treatment for chronic and acute metal poisoning. Chelators include d-penicillamine, calcium disodium edetate (CaNa_2_ EDTA), British antilewisite (BAL), and Cuprimine (Jang and Hoffman, 2011; Cavalu et al., 2020). However, chelators can cause side effects such as headaches, weariness, renal failure, nasal congestion, and life-threatening hypocalcemia (Jang and Hoffman, 2011). The most important considerations when performing chelation therapy are the specificity and dosage of the chelators.

The identification of individual metal exporters has led to the development of innovative therapies for metal-induced neurotoxicity. Mutations in the newly discovered manganese transporter *SLC30A10* cause dystonia, hypermanganesemia, parkinsonism, and manganism, a condition characterized by the release of too much intracellular manganese (Choi et al., 2007; Quadri et al., 2012; Leyva-Illades et al., 2014; Chen et al., 2015b). While a comprehensive investigation of its substrates has not yet been completed, it appears to only transport manganese (Leyva-Illades et al., 2014; Chen et al., 2015a). Pharmaceuticals containing compounds that enhance SLC30A10 export stimulated manganese efflux but have little effect on other metals. The risks of chelation can be greatly reduced using this technique. However, the specific transporters for different metals, such as SLC30A10 for manganese, remain largely unknown and must be exhaustively studied in the future to enable the development of new therapies for metal-induced toxicity.

## Metal exposure on public health: Low and middle-income countries

The most significant natural resource is water. Surface water samples from the Rupsariver in Bangladesh were taken in the summer and winter seasons, and pH, electrical conductivity (EC), chromium, nickel, copper, arsenic, cadmium, and lead levels were determined to assess the risk of metal toxicity, identify its potential sources, and predict health risk from metals in water. Their average concentrations and standard deviations in the summer season were determined to be: chromium (7.20 ± 0.613 g/L), lead (7.09 ± 0.904 g/L), arsenic (5.45 ± 0.441 g/L), copper (5.36 ± 0.471 g/L), nickel (3.85 ± 0.694 g/L), and cadmium (0.975 ± 0.106 g/L). Similarly, in the winter season, they were determined to be: chromium (8.87 ± 0.756 g/L), lead (7.32 ± 0.93 g/L), arsenic (6.05 ± 0.490 g/L), copper (6.02 ± 0.529 g/L), nickel (5.48 ± 0.986 g/L), and cadmium (1.38 ± 0.151 g/L). Methods such as correlation analysis, principal component analysis (PCA), and cluster analysis (CA) were used to identify the source of harmful metals in the water [1]. Except for copper, total heavy metal toxicity load and heavy metal evaluation index values exceeded permitted levels. During both seasons, 85% of the total samples were determined to pose moderate ecological risks, while the remaining 15% posed low ecological risks, based on the ecological risk index classification. Oral exposure revealed a high non-carcinogenic risk for a single element (Proshad et al., 2021). The oral exposure hazard index values were 4.17 for adult males, 3.67 for adult females, and 8.64 for children, indicating that non-carcinogenic effects are likely. The carcinogenic risks of nickel and arsenic from regular oral and dermal contact were higher than the standard value (>1.0 × 10^–4^), indicating potential cancer risks to adult males and females and children in the studied area (Proshad et al., 2021).

Around 25% of global fatalities and disorders are caused by harmful environmental exposures (Heng et al., 2022). Metals are a common source of dangerous environmental exposure, with levels regularly exceeding the recommended limits. Currently, 632 million children in lower-middle-income countries (LMICs) have blood lead levels above both the US Centers for Disease Control’s former public health action standard of 5 μg/dl (Ericson et al., 2021) and the updated 2021 standard of 3.5 μg/dl (Sink, 2022). Manganese levels in drinking water surpass 400 μg/L in over 50 nations, including Bangladesh, Cambodia, Egypt, and Ghana. While the World Health Organization (WHO) no longer provides manganese guidelines (Frisbie et al., 2012), studies have indicated that excessive levels are associated with a variety of adverse effects, including impaired intellectual performance in children (Ericson et al., 2007). More than 4.5 million people in Latin America are chronically exposed to arsenic in their drinking water, in some cases more than 200 times higher than the limit of 10 μg/L set by WHO (McClintock et al., 2012).

Many heavy metals pollute the air, water, and soil environment due to their unregulated use in agriculture, mining, smelting, illegal refining, and industrial production. Because they are non-biodegradable, their environmental concentrations may gradually increase over time. Consequently, cumulative human population exposures can have long-term consequences (Tchounwou et al., 2012). Children are more vulnerable to the negative health impacts of environmental exposures because of their rapid neurodevelopment. The brain is most plastic during the first 1,000 days of life, during which it undergoes a series of complex processes such as neurogenesis, myelination, and synaptic pruning (Grantham-McGregor et al., 2007). These processes build over time, leading to important cognitive functions such as language and speech, attention, conduct, and reasoning (Villar et al., 2019). Therefore, perturbations in their biological environment, such as toxicant exposure, can disrupt the precise orchestration of events, resulting in irreversible downstream effects such as neurodevelopmental delays, behavioral issues, and learning difficulties (Davis et al., 2019). In addition to this stage of fast brain plasticity, children are more vulnerable to adverse environmental exposures than adults due to additional biological and social reasons. Children consume more food and water in proportion to their body weight, spend more time on the ground and the floor, and eat more non-food things (Mamtani et al., 2011).

The negative impacts of heavy metals on child neurodevelopment have been extensively studied in high-income countries (HICs). Lead is the heavy metal with the greatest evidence of neurodevelopmental harm, with a dose-dependent drop in IQ scores (Searle et al., 2014). Even with blood lead levels below five ug/dL, the National Toxicology Program found the data sufficient to detect the detrimental effects of lead on cognition and heightened attention-related and other problematic behaviors (Budtz-Jørgensen et al., 2013). Other metals, such as arsenic, cadmium, manganese, and mercury, have also been found to negatively impact several aspects of neurodevelopment, including cognition and behavior (Ericson et al., 2007; Jett et al., 2020). There is inconclusive evidence about the impact of prenatal arsenic exposure on neurodevelopment (Freire et al., 2018; Jiang et al., 2018). However, the majority of studies evaluating the association between heavy metals and infant neurodevelopment take place in HICs rather than LMICS, which account for 90% of children worldwide (Husain et al., 2021). An estimated 43% of children in LMICs do not reach their full neurodevelopmental potential due to various factors, including environmental exposures (Lu et al., 2016). This inability to realize their full potential influences their quality of life and economic potential, highlighting the importance of understanding the effects of metals on neurodevelopment in LMICs environments (Grantham-McGregor et al., 2007).

## Conclusion and future prospects

An imbalance of metal ions is initiated or mediated by a cascade of processes that inevitably leads to neural network dysfunction in many NDDs, including oxidative stress, protein misfolding and aggregation, mitochondrial dysfunction, and energy depletion. The fundamental mechanisms underlying some of these activities remain unknown, and how ion homeostasis is maintained and disrupted in the brain is becoming a contentious issue. The importance of inadequately liganded metals has been overlooked in the past. A better understanding of the multiple factors implicated in these processes is crucial for determining the pathophysiological mechanisms underlying the abundance of metal ions and developing therapeutic approaches that can disrupt the chain of pathological events that occur in many NDDs, including AD, the etiology of which remains unknown for many. It remains unclear whether different metals, such as iron, zinc, copper, and aluminum, have similar or dissimilar modes of action. Understanding these mechanisms requires a multisystem integrative approach, leading to future advancements in neurodegenerative research.

The intricate association between biometal metabolism, genetic and environmental exposures, and the pathophysiology of NDDs merits additional exploration, particularly in light of recent developments in metal neurobiology. While the corroboration from experimental and transgenic animal studies is compelling, when combined with findings for the human brain, they suggest that metal ions play a significant role in neurodegeneration but do not provide conclusive answers on the causality of metal-related processes or efficacious preventive and therapeutic approaches in humans. Metalloproteomics developments have contributed to improved knowledge of the mechanics and exact involvement of metalloenzymes and proteins in the brain (Lothian et al., 2013).

There is little that can be done to slow the progression of neurodegeneration at present. Its multifaceted presentation requires a fundamental change toward developing multidrug treatments. Metal ions are implicated in the majority of these degenerative disorders, making them a promising target for future therapeutic approaches. One strategy is to chelate and sequester the ions, limiting their ability to interfere with protein folding or prevent them from undergoing oxidative processes (Fasae et al., 2021). Redistributing metal ions with newer approaches has therapeutic effects. Recent research suggests that treating cellular copper shortage may help to prevent neurodegeneration (Gromadzka et al., 2020). Similarly, iron-chelating drugs such as hydroxypyridones may facilitate the redistribution of iron through mobilization with transferrin to treat several NDDs, including AD (Singh et al., 2019). Many drugs have recently been developed to reduce metal ions associated with both metal-induced Aβ aggregation and ROS generated by this and other aggregates through chelation. Developing drugs with a multitargeted action may be the next step in treating NDDs such as AD (Sales et al., 2019), but they will need to be validated and evaluated further.
